# Discovery of Farnesoid X Receptor Antagonists Based on a Library of Oleanolic Acid 3-*O*-Esters through Diverse Substituent Design and Molecular Docking Methods

**DOI:** 10.3390/molecules22050690

**Published:** 2017-04-26

**Authors:** Shao-Rong Wang, Tingting Xu, Kai Deng, Chi-Wai Wong, Jinsong Liu, Wei-Shuo Fang

**Affiliations:** 1 State Key Laboratory of Bioactive Substances and Functions of Natural Medicines, Institute of Materia Medica, Chinese Academy of Medical Sciences & Peking Union Medical College, 2A Nanwei Road, Beijing 100050, China; wangshaorong@outlook.com (S.-R.W.); dkatalent@sina.com (K.D.); 2 Center for Drug Evaluation, China Food and Drug Administration, 1A Fuxing Road, Beijing 100038, China; 3 School of Life Sciences, University of Science and Technology of China, Hefei 230026, China; xu_tingting@gibh.ac.cn; 4 State Key Laboratory of Respiratory Disease, Guangzhou Institutes of Biomedicine and Health, Chinese Academy of Sciences, Guangzhou 510530, China; liu_jinsong@gibh.ac.cn; 5 NeuMed Pharmaceuticals Limited, Unit 509, 5/F BioTech Center I, No. 9 Science Park West Avenue, Shatin, Hong Kong, China; wongcw123456@yahoo.com

**Keywords:** FXR antagonist, oleanolic acid, compound diversity, library design, molecular modeling

## Abstract

The pentacyclic triterpene oleanolic acid (OA, **1**) with known farnesoid X receptor (FXR) modulatory activity was modified at its C-3 position to find new FXR-interacting agents. A diverse substitution library of OA derivatives was constructed in silico through a 2D fingerprint similarity cluster strategy. With further docking analysis, four top-scored OA 3-*O*-ester derivatives were selected for synthesis. The bioassay results indicated that all four compounds **3** inhibited chenodeoxycholic acid (CDCA)-induced FXR transactivation in a concentration-dependent mode. Among them **3b** and **3d** are more active than the parent compound OA. A molecular simulation study was performed to attempt to explain the structure-activity relationship (SAR) and the antagonistic action. To the best of our knowledge, this is the first report on semi-synthetic pentacyclic triterpenoids with FXR-modulatory activities.

## 1. Introduction

Farnesoid X receptor (FXR), initially discovered as a bile acid homeostasis regulating transcriptional factor [1,2], is well known for its involvement in diverse metabolic processes, including the control of cholesterol, lipid and glucose metabolism [3]. This has made FXR an attractive pharmacological target, especially for the treatment of cholestasis, atherosclerosis and diabetes, etc. In addition, FXR has also been found to play a role beyond metabolism, e.g., in tumorigenesis [4].

**Figure 1:**
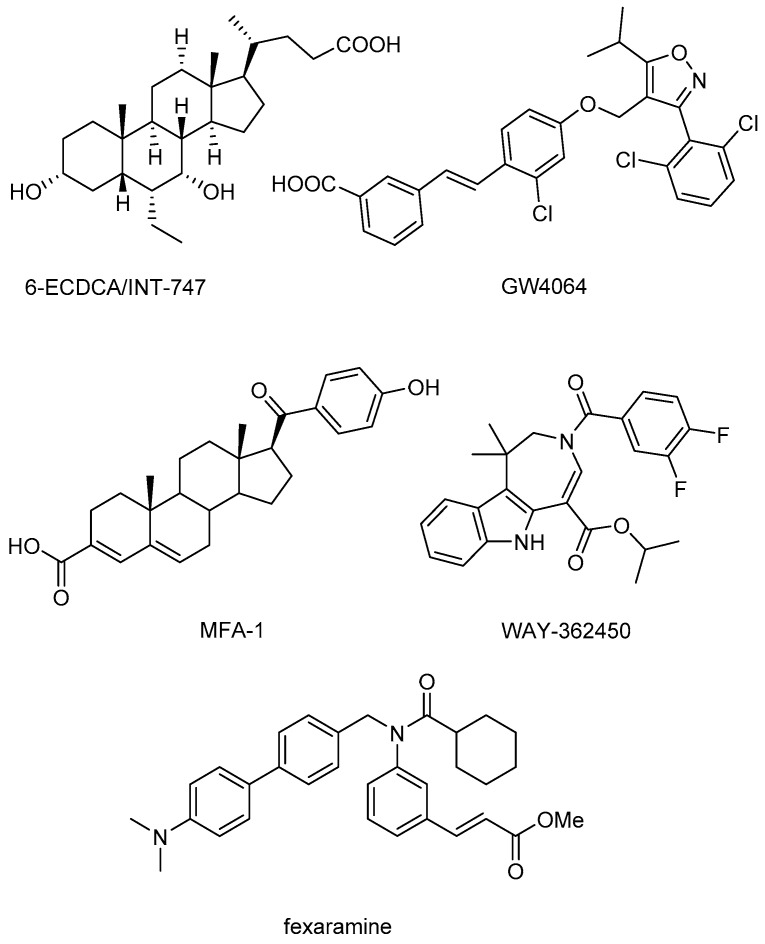
Structures of some farnesoid X receptor (FXR) agonists.

Many medicinal chemistry efforts have been made to prepare FXR agonists [5,6] to activate the transcription of various genes under the control of FXR. Some of them are very potent FXR-interacting agents with sub-μM or even nM level IC_50_ values, such as 6-ECDCA/INT-747, the first FXR agonist approved by the U.S. FDA in 2016 for the treatment of primary biliary cholangitis, GW4064, feraxamine, MFA-1 and WAY-362450 (Figure 1), etc. To date, aside from the success of 6-ECDCA/INT-747, only a limited number of these agonists have however been studied in humans.

It was reported that full agonism of FXR may lead to the activation of various downstream genes, resulting in complex and unexpected biological responses. For example, some complete FXR agonists were found to decrease the level of high-density lipoprotein (HDL) while increasing the level of low-density lipoprotein (LDL) [7], which may lead to cardiovascular-related adverse effects. In this regard, the exploration of partial agonists, antagonists, or modulators has been proposed as an alternative.

In recent years, some FXR antagonists have displayed beneficial effects in cholestasis [8] and hypercholesterolemia [9] animal models, suggesting that FXR antagonists may be worth pursuit in this context. However, the available pharmacological data on FXR antagonists are still limited and some results are contradictory, therefore, we tried to discover more antagonists to increase the structural and also possibly functional diversity.

**Figure 2:**
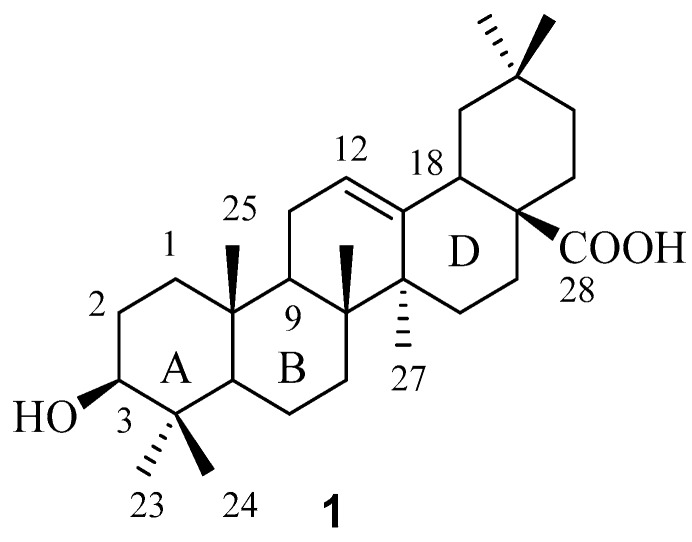
Structure of oleanolic acid (OA).

In 2010, one of our co-authors reported oleanolic acid (Figure 2, OA, **1**) as a selective FXR modulator [10]. It was found that, OA dose-dependently suppressed the activity of FXR-LBD (ligand binding domain) induced by its endogenous ligand, chenodeoxycholic acid (CDCA). At 25 μM concentration, it partially blocked CDCA induction of bile salt export protein (BSEP), but did not affect the expression of organic solute transporter (OST-β). The expression of short heterodimer partner (SHP) was slightly enhanced and its induction by CDCA was not affected in the presence of OA, and the expression levels of cholesterol 7α-hydroxylase (CYP7A1) was suppressed in the presence of CDCA, OA, and their combination. Therefore, OA functioned as a gene selective modulator of FXR in HepG2 cells. However, since both SHP and CYP7A1 are regulated by many different signaling pathways, including several nuclear receptors, and the effects on SHP or CYP7A1 cannot unambiguously be interpreted as activity on FXR [11]. In contrast, BSEP is a more specific target gene which is almost exclusively regulated by FXR. From this perspective, OA may better be regarded as a partial antagonist of FXR. In addition, a low dose of OA (20 mg/kg) could protect against lithocholic acid (LCA)-induced cholestasis in mice [12], and attenuate obstructive cholestasis in bile duct-ligated mice [13,14], partially due to its FXR antagonism.

Although OA has not been studied extensively, these preliminary findings indicate that it could be used as a lead compound for further optimization. To the best of our knowledge, only one natural analogue of OA, platycodin d (a 3,28-substituted saponin), has been known to exert weak activity on FXR [15]. In addition, 3-β-(2-carboxybenzoyloxy)-oleanolic acid (NPLC441) was previously reported to bound strongly to retinoid X receptor-LBD [16]. Based on the above information, we chose to derive 3β-OH during our initial effort to optimize OA.

## 2. Results and Discussion

In order to maximize our chance of finding better FXR-interacting compounds during our exploration in the chemical space around the C-3 position in OA, we decided to introduce diverse substituents, among which a limited number of representative structures were selected with the aid of different filters, for this position.

To build up a small diverse OA 3-*O*-ester library consisting of various carboxyl acids from a commercial compounds database, MDL^®^ Available Chemicals Directory (ACD) containing a total of 34,895 carboxylic acids was utilized. Non-compliant compounds according to Linpinski’s “Rule of 5” [17] were first filtered out of the ~35 K acids in the library to minimize the total number of the building blocks by using the Discovery Studio 2.0 software (Accelrys Inc., San Diego, CA, USA). Considering the frequently observed violation of this empirical rule by natural products, and also with the intention of including the physiochemical properties of the parent compound **1** itself in the parameter settings, we modified this rule with our own customized criteria:

(1) For a balance between more choice of building blocks and drug-like properties, i.e., not too large a molecular weight (MW), we set the upper MW limit of the carboxylic acids to 200. In Lipinkski’s rule, the MW is set to below 500 for orally active compounds. Considering the MW of **1** itself is 456, using MW 500 for 3-*O*-esters of OA would significantly limit the choice of carboxylic acids.
(2) For other parameter settings, e.g., hydrogen bond donors (HBD) and hydrogen bond acceptors (HBA), the functional groups presenting already in OA (at C-3 and C-28) are also considered, so these parameters were set as follows: HBD equal or less than 4, HBA equal or less than 8, the number of rotatable bonds equal or less than 7, the number of rings equal or less than 2, the number of aromatic rings equal or less than 2.

After filtering the ~35 K acids using the above criteria, only 6106 compounds were left in the library. Next, the compounds with highly reactive or unstable groups, such as aminonitrile, thionitrile, epoxide, aziridine, disulfide, nitrogen oxides, azo compounds, β-lactams, etc., were also filtered out. In addition, the molecules which seemed not to be very “drug-like”, such as long-chain fatty acids, gem-dicarboxylic acids, compounds with boron, selenium or silicon atoms, ion exchange resins, quaternary ammonium salt, etc., as well as inorganic or organic salts of some carboxylic acids were also removed. After all the above filtering steps, 4454 compounds remained and were subjected to the diversity analysis.

A 2D fingerprint [18] is a set of binary strings encoding the presence or absence of sub-structural fragments in a certain molecule, which can be used for the measurement of inter-molecular structural similarity and for the evaluation of structural and biological diversity of a compound library. We chose molecular fingerprint FCFP_4 as the descriptor and clustered all of the 4454 carboxylic acids to 285 clusters using Accelrys Discovery Studio 2.0. One acid was randomly selected from each cluster to assemble a sub-library of 285 acids. Those acids were used as the building blocks to create a 3-*O*-ester library of OA, for the next step virtual screening by docking.

**Figure 3:**
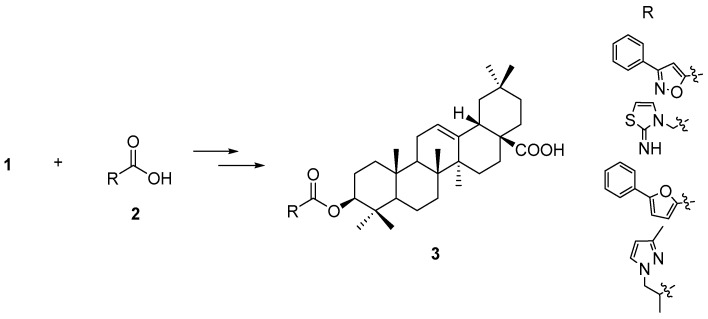
OA analogs to be synthesized.

A total of 285 OA 3-*O*-esters and one positive control molecule (fexaramine, Figure 1) were screened against FXR (PDB code 1OSH) using Autodock 4.2 (The Scripps Research Institute, La Jolla, CA, USA). The positive control fexaramine scored the highest among those compounds with a p*K*_i_ of 10.39, and its binding mode is consistent with the experimental determination. Further examination of the positive control and other four top-scored compounds **3a**–**d** (Figure 3) using the Autodock Vina software showed a similar scoring list and binding position in FXR compared with the docking results from Autodock 4.2. The best compound in the triterpenoid library has a p*K*_i_ of 9.68 while the p*K*_i_ values of the other three compounds ranged from 8.00 to 9.35 (Table 1).

**Scheme 1:**
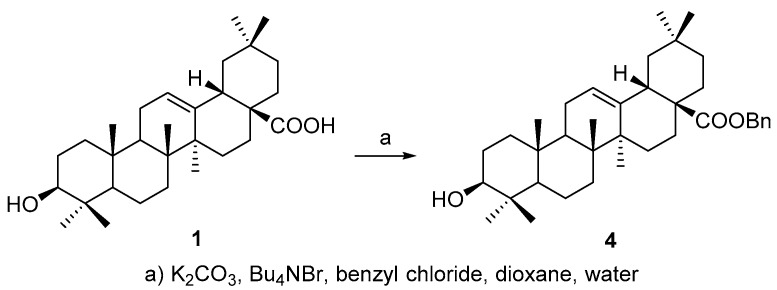
Preparation of compound **4**.

All above four triterpenoids were synthesized as below. The C-28 carboxyl group was protected before C-3 derivatization, according to a modified procedure of literature [19], to afford benzyl 3β-hydroxyolean-28-oate (**4**) (Scheme 1) so as to improve the overall yield of the OA 3-*O*-esters, based on our experience on triterpene chemistry.

**Scheme 2:**
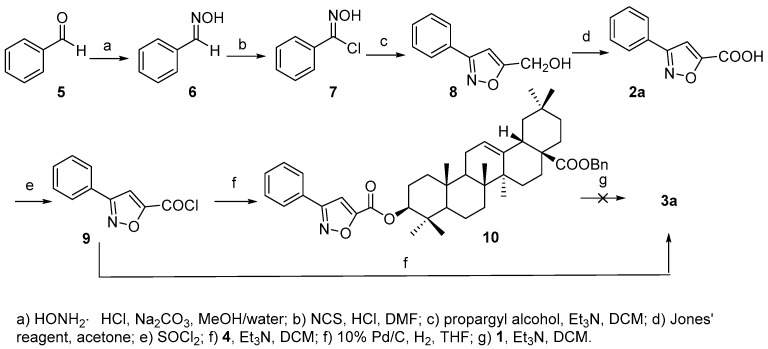
Synthesis of compound **3a**.

Compound **3a** was prepared as shown in Scheme 2. Acid **2a** was first synthesized according to [20]. Starting from benzaldehyde (**5**), consequential transformations including a [3 + 2] cycloaddition of chlorobenzaldoxime (**7**) and propargyl alcohol to afford phenyl substituted isoxazole acid **2a**, which was further converted to acyl chloride **9** that reacted with **4** to furnish the 3-*O*-acylated analogue **10**. However, the debenzylation of **10** to afford the desired product **3a** proved troublesome, maybe due to the ring-opening of the isoxazole group under hydrogenolysis conditions as reported [21]. Thus, **3a** was finally prepared in reasonable yield (63.0%) by direct acylation of **1** with **9**.

**Scheme 3:**
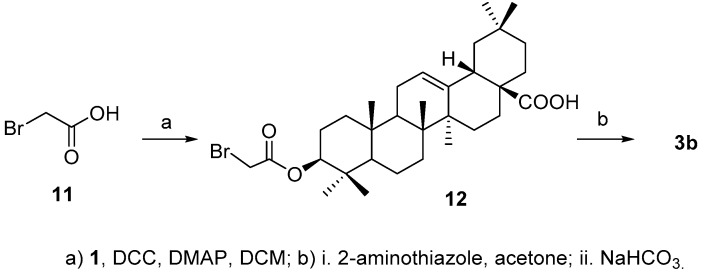
Synthesis of compound **3b**.

For the synthesis of **3b**, acid **2b** was initially prepared successfully by reacting 2-aminothiazole with bromoacetic acid (**11**) [22], but the aminothiazole acetic acid failed to attach to 3-OH in **4** either by coupling reaction in the presence of *N*,*N*′-dicyclohexylcarbodiimide (DCC) or *N*-(3-dimethylaminopropyl)-*N*′-ethylcarbodiimide (EDCI) in the presence of 4-dimethylaminopyridine (DMAP) or using the corresponding acyl chloride. Alternatively, bromoacetic acid **11** was coupled with **1** to afford **12** [23], which was then treated with 2-aminothiazole to afford the desired product **3b** (Scheme 3) in 50.6% yield over two steps.

**Scheme 4:**
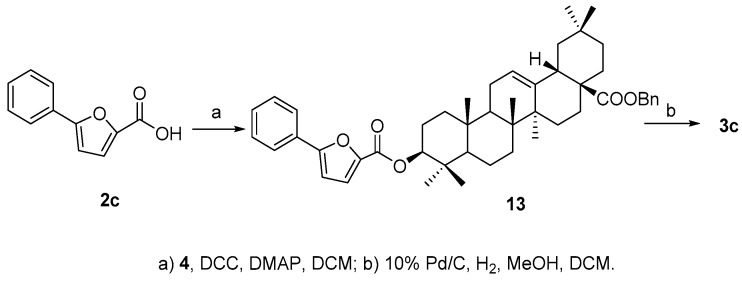
Synthesis of compound **3c**.

Compound **3c** was much easier to synthesize since acid **2c** is commercially available. Ester **13** was obtained by coupling of **2c** and **4** in the presence of DCC and DMAP, and then the benzyl group was removed readily by catalytic hydrogenolysis to afford the final product **3c** in good overall yield (89.6%) (Scheme 4).

**Scheme 5:**
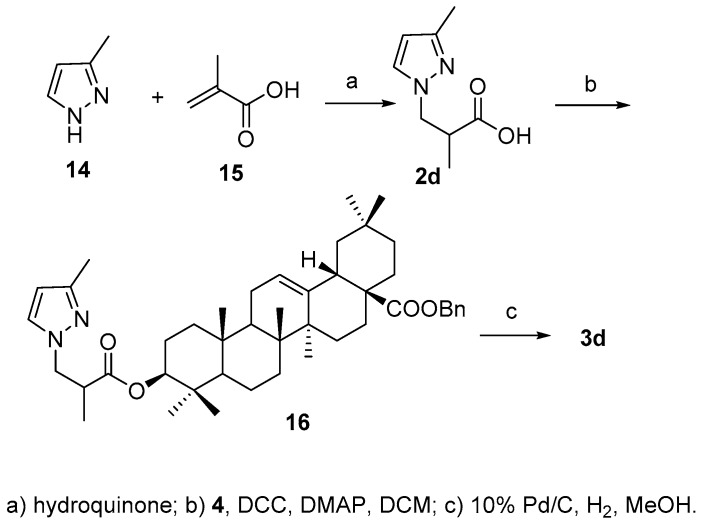
Synthesis of compound **3d**.

According to the literature method [24], acid **2d** was synthesized by Michael addition reaction between 3-methylpyridine (**14**) and α-methylacrylic acid (**15**) in the presence of hydroquinone in 81.0% yield., After attachment of acid to 3-OH and debenzylation of 28-COOH, the final product **3d** was obtained in 41.6% yield over two steps (Scheme 5).

The biological activities of these OA analogues against FXR were evaluated using a co-transfection assay in human embryonic kidney 293T cells. None of the compounds had any effect when incubated with the cells alone, but they could inhibit CDCA-induced FXR transactivation in a concentration-dependent manner. The IC_50_ values of compounds **3a**–**d** were measured as 19.41, 7.03, 13.74, and 9.03 μM, respectively (Table 1). It is worth noting that the trend of measured IC_50_ values was consistent with the predicted p*K*_i_ from the virtual screening, indicating the feasibility of this ligand-FXR model.

**Figure 4:**
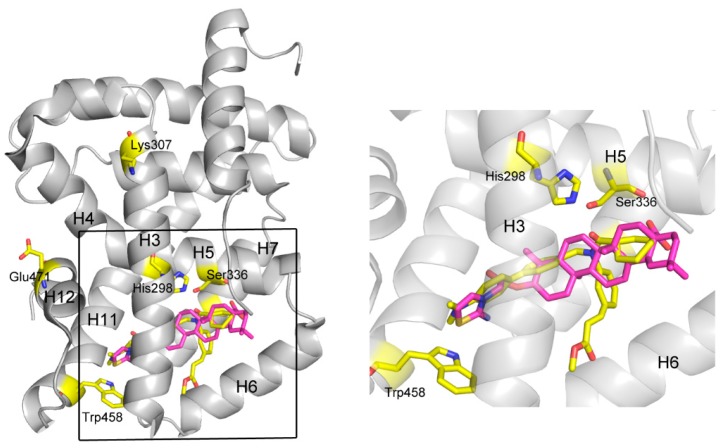
Binding modes of fexaramine (yellow) and compound **3b** (magenta) into FXR. Key residues involved in the formation of “charge clamp” and the interactions with fexaramine and compound **3b** are all marked and presented as sticks. Helices discussed in the text are numbered.

A further, more exhaustive docking was carried out to study the mechanism of action for these ligands by docking them into FXR (PDB code 1OSH) using Autodock 4.2. For **3b**, it was shown that two pairs of T-shaped π-π interactions exist between the thiazoline ring and one aromatic residue (Trp458, H11, Figure 4). This interaction seems important for anchoring the compound in the binding site, since other atoms of **3b** contribute to interactions only by shape matching and hydrophobic interaction with H3 (H=helix), H5, H6, and H7. It is believed that H12 plays a crucial role in the activation of FXR by both natural or unnatural agonists. Agonists such as CDCA could activate FXR by directly stabilize H12 through forming two important H-bonds with the protein [25] However, in some cases, not only H12 but also the other helices, especially H3, are necessary to enable coactivator recruitment [26]. A so-called “charge clamp”, formed by a glutamic acid residue from H12 (Glu471 in FXR) and a lysine residue from H3 (Lys307 in FXR) in the coactivator-interacting surface, exists in many NRs and is able to stabilize their active conformations [27]. In addition, there are a significant number of contacts between the methyl ester group in fexaramine and H3/H6 of FXR, which significantly contribute for its potency [28]. In contrast, these interactions are absent in the model of **3b** binding to FXR, although the binding positions of these two compounds are similar. Moreover, the amide CO of fexaramine formed two H-bonds with His298 in the middle section of H3 and with Ser336 in H5, respectively, strengthening the stabilizing effect of the ligand. Although there is a hydrophobic interaction between compound **3b** and His298, it might not be strong enough to stabilize the overall conformation of H3, especially hold the remote key residue Lys307 (one of the component of the “charge clamp”) in the proper position, thus compromising the whole stabilizing effect of the compound. The binding mode of compound **3d** was very similar with **3b**, so that they have similar activities. The lack of some important interactions, such as the H-bonds with His298 and Ser336, and the hydrophobic interaction with H3/H6, may account for their inability to exert agonist activity, so that they exhibited only antagonistic effect.

In contrast, the binding poses for the other analogs are different. For OA, since the molecule is smaller than other derivatives, there is lack of some interactions with H5 and H6 in FXR (Figure S1). However, an existing H-bond between C-28 COOH of OA and Tyr373 at H7 might compensate some lost interactions. For **3a** (Figure S1, **3c** is similar), although there is an H-bond between this compound and Tyr373 in a subpocket, which is encompassed by H3, H11, and H12, the binding pose and position are quite different from those of the above mentioned trierpenoids and fexaramine. Thus, the binding affinities of compounds OA, **3a**, and **3c**, which are lower than the more active compounds **3b** and **3d**, made them less potent for the FXR modulation.

## 3. Materials and Methods

### 3.1. General Information

Oleanolic acid (purity > 98%) was purchased from National Institute for the Control of Pharmaceutical and Biological Products (Beijing, China). All chemical reagents were of standard quality and used without further purification. Column chromatography was carried out with silica gel as the stationary phase. ^1^H- and ^13^C-NMR spectra were measured on Mercury-500 or Mercury-300 spectrometers (Varian, Palo Alto, CA, USA). The mass spectra (MS) were measured on an 1100 LC/MSD high performance ion trap mass spectrometer and an ESI mass spectrometer (LCQ) (Agilent, Santa Clara, CA, USA).

### 3.2. Synthesis of Compound ***10***

To a stirred solution of **4** (20 mg, 0.036 mmol) in dry dichloromethane (DCM) (0.5 mL) was added Et_3_N (15.6 μL, 0.108 mmol) and a solution of **9** (0.072 mmol) in dry DCM (0.5 mL) under Ar. The mixture was stirred for 3 h at room temperature, then DCM (10 mL) was added, and the organic layer was washed with 2 M HCl (aq.) (5 mL × 3), sat. NaHCO_3_ (aq.) (5 mL × 3) and sat. NaCl (aq.) (5 mL × 3) sequentially, dried over Na_2_SO_4_ and concentrated. The residue was purified through a silica chromatography column (petroleum ether−EtOAc 20:1) to afford the product **10** as a white solid (16 mg, 61.5%). ^1^H-NMR (300 MHz, CDCl_3_): *δ* 7.84 (2H, m, H-2, 6 of Ph), 7.48 (3H, m, H-3, 4, 5 of Ph), 7.35 (5H, m, Bn), 7.20 (1H, s, H of isoxazole), 5.30 (1H, br s, H-12), 5.08 (2H, ABq, *J* = 12.6 Hz, CH_2_ of Bn), 4.79 (1H, t, *J* = 8.1 Hz, H-3α), 2.92 (1H, d, *J* = 9.6 Hz, H-18), 1.14 (3H, s, CH_3_), 1.00 (3H, s, CH_3_), 0.96 (6H, s, 2CH_3_), 0.93 (3H, s, CH_3_), 0.91 (3H, s, CH_3_). ESI-MS: *m*/*z* [M + H]^+^ 718.7.

### 3.3. Synthesis of Compound ***3a***

To a stirred solution of **1a** (25 mg, 0.055 mmol) in dry DCM (0.5 mL) was added Et_3_N (23.8 μL, 0.165 mmol) and a solution of **9** (0.11 mmol) in dry DCM (0.5 mL) under Ar. The mixture was stirred for 10 h at room temperature, then DCM (10 mL) was added, and the organic layer was washed with 2 M HCl (aq.) (5 mL × 3), sat. NaHCO_3_ (aq.) (5 mL × 3) and sat. NaCl (aq.) (5 mL × 3) sequentially, dried over Na_2_SO_4_ and concentrated. The residue was purified through a silica chromatography column (petroleum ether−EtOAc−HOAc 15:1:0.1) to afford the product (**3a**) as a white solid (19 mg, 63.0%). ^1^H-NMR (300 MHz, CDCl_3_): *δ* 7.84 (2H, m, H-2, 6 of Ph), 7.48 (3H, m, H-3, 4, 5 of Ph), 7.21 (1H, s, H of isoxazole), 5.30 (1H, brs, H-12), 4.80 (1H, t, H-3α), 2.83 (1H, d, *J* = 12 Hz, H-18), 1.16 (3H, s, CH_3_), 1.01 (3H, s, CH_3_), 1.00 (3H, s, CH_3_), 0.97 (3H, s, CH_3_), 0.94 (3H, s, CH_3_), 0.92 (3H, s, CH_3_), 0.79 (3H, s, CH_3_). ^13^C-NMR (125 MHz, CDCl_3_): *δ* 183.8, 163.0, 161.3, 156.7, 143.6, 130.5, 129.1 (2C), 128.1, 126.9 (2C), 122.5, 107.1, 83.6, 55.3, 47.6, 46.5, 45.8, 41.6, 40.9, 39.3, 38.1, 37.0, 33.8, 33.1, 32.5, 32.4, 30.7, 28.2, 27.7, 25.9, 23.6, 23.5, 23.4, 22.9, 18.2, 17.2, 16.8, 15.4. ESI-MS: *m/z* [M − H]^−^ 626.4.

### 3.4. Synthesis of Compound ***12***

To a stirred solution of **1a** (30 mg, 0.066 mmol) in DCM (1 mL) was added bromoacetic acid (18 mg, 0.13 mmol), DCC (54 mg, 0.26 mmol) and DMAP (0.8 mg, 0.0066 mmol). Then, the mixture was stirred at room temperature for 1 h. Then the precipitate was filtered and the filtrate was concentrated. The residue was purified through a silica chromatography column (petroleum ether−EtOAc 20:1) to afford the product (**12**) as a white solid (35 mg, 92.0%). ^1^H-NMR (300 MHz, CDCl_3_): *δ* 5.27(1H, brs, H-12), 4.55 (1H, t, *J* = 8.1 Hz, H-3α), 3.82 (2H, ABq, *J* = 12.3 Hz, CH_2_), 2.81 (1H, dd, H-18), 1.18 (3H, s, CH_3_), 0.94 (3H, s, CH_3_), 0.92(3H, s, CH_3_), 0.90 (6H, br s, 2CH_3_), 0.88 (3H, s, CH_3_), 0.79 (3H, s, CH_3_). ESI-MS: *m/z* [M + H]^−^ 577.4.

### 3.5. Synthesis of Compound ***3b***

To a stirred solution of **12** (35 mg, 0.06 mmol) in acetone (1.5 mL) was added 2-aminothiazole (18 mg, 0.18 mmol). The mixture was stirred at 50 °C for 20 h, then DCM (20 mL) and NaHCO_3_ (aq.) (10 mL) were added. And the mixture was stirred for another 30 min at room temperature. Then the aqueous layer was extracted with DCM (5 mL × 3). The organic layer was collected, dried over Na_2_SO_4_ and concentrated. The residue was purified through a silica chromatography column (CHCl_3−_MeOH−Et_3_N 25:1:0.2) to afford the product (**3b**) as a white solid (20 mg, 55.0%). ^1^H-NMR (500 MHz, C_5_D_5_N): *δ* 6.25 (1H, d, *J* = 5.0 Hz, H of thiazolimine), 5.96 (1H, d, *J* = 5.0 Hz, H of thiazolimine), 5.44 (1H, br s, H-12), 4.83 (2H, q, *J* = 10.2 Hz, CH_2_), 4.75 (1H, dd, *J* = 5.0, 12.0 Hz, H-3α), 3.33 (1H, d, *J* = 10.5 Hz, H-18), 1.24 (3H, s, CH_3_), 0.99 (3H, s, CH_3_), 0.94 (9H, br s, 3CH_3_), 0.85 (3H, s, CH_3_), 0.77 (3H, s, CH_3_). ^13^C-NMR (125 MHz, C_5_D_5_N): *δ* 180.3, 168.9, 163.0, 144.9, 128.3, 122.3, 97.4, 82.1, 55.4, 47.8, 47.3, 46.7, 46.5, 42.1, 42.0, 39.7, 38.1, 38.0, 37.1, 34.2, 33.3, 33.2, 33.0, 31.0, 28.3, 28.2, 26.2, 23.8, 23.7, 23.7, 18.4, 17.4, 16.9, 15.3. ESI-MS: *m/z* [M − H]^−^ 595.5.

### 3.6. Synthesis of Compound ***13***

To a stirred solution of **4** (40 mg, 0.07 mmol) in DCM (1 mL) was added **2c** (55 mg, 0.29 mmol), EDCI (70 mg, 0.37 mmol) and DMAP (5 mg, 0.04 mmol). The mixture was stirred at 50 °C for 4 h. Then the reaction solution was concentrated. The residue was purified through a silica chromatography column (petroleum ether−EtOAc 20:1) to afford the product (**13**) as white solid (41 mg, 78.0%). ^1^H-NMR (300 MHz, CDCl_3_): *δ* 7.78 (2H, d, *J* = 7.5 Hz, H-2, 6 of Ph) 7.42 (3H, m, H-3, 4, 5 of Ph), 7.34 (5H, s, Bn), 7.10 (1H, d, *J* = 1.8 Hz, H of furan), 6.73 (1H, d, *J* = 1.8 Hz, H of furan), 5.30 (1H, br s, H-12), 5.08 (2H, ABq, *J* = 12.0 Hz, CH_2_ of Bn), 4.72 (1H, t, *J* = 8.7 Hz, H-3α), 2.91 (1H, d, *J* = 12.9 Hz, H-18), 1.14 (3H, s, CH_3_), 0.99 (3H, s, CH_3_), 0.95 (6H, br s, 2CH_3_), 0.92 (3H, s, CH_3_), 0.90 (3H, s, CH_3_), 0.63 (3H, s, CH_3_). ESI-MS: *m/z* [M + Na]^+^ 739.6.

### 3.7. Synthesis of Compound ***3c***

To a stirred solution of **13** (41 mg, 0.057 mmol) in a mixed solvent of MeOH (0.4 mL) and DCM (0.1 mL) was added 10% Pd/C (4 mg) and H_2_. The mixture was stirred for 20 h at room temperature, then filtered and the filtrate was concentrated. The residue was purified through a silica chromatography column (petroleum ether−acetone 15:1) to afford the product (**3c**) as a white solid (29 mg, 89.6% based on 4 mg recovery of substrate). ^1^H-NMR (300 MHz, CDCl_3_): *δ* 7.78 (2H, d, *J* = 7.5 Hz, H-2, 6 of Ph), 7.41 (2H, t, H-3, 5 of Ph), 7.33 (1H, t, H-4 of Ph),7.20 (1H, d, *J* = 3.3 Hz, H of furan), 6.73 (1H, d, *J* = 3.6 Hz, H of furan), 5.29 (1H, br s, H-12), 4.74 (1H, t, H-3α), 2.83 (1H, d, *J* = 10.5 Hz, H-18), 1.16 (3H, s, CH_3_), 1.00 (6H, br s, 2CH_3_), 0.96 (3H, s, CH_3_), 0.94 (3H, s, CH_3_), 0.92 (3H, s, CH_3_), 0.78 (3H, s, CH_3_). ^13^C-NMR (75 MHz, CDCl_3_): *δ* 184.1, 158.7, 157.4, 144.1, 143.6, 129.6, 128.8 (3C), 124.8 (2C), 122.6, 119.4, 106.7, 81.5, 55.3, 47.5, 46.5, 45.8, 41.6, 40.9, 39.3, 38.1, 37.0, 33.8, 33.1, 32.5, 32.4, 30.7, 28.2, 27.7, 25.9, 23.6, 23.4, 22.9, 18.2, 17.2, 16.8, 15.4. ESI-MS: *m/z* [M − H]^−^ 625.5.

### 3.8. Synthesis of Compound ***16***

To a stirred solution of **4** (10 mg, 0.018 mmol) in DCM (0.4 mL) was added **2d** (6 mg, 0.036 mmol), DCC (14.8 mg, 0.072 mmol) and DMAP (0.2 mg, 0.0018 mmol). The mixture was stirred for 2 h at room temperature. Then the precipitate was filtered and the filtrate was concentrated. The residue was purified through a silica chromatography column (petroleum ether−EtOAc 20:1) to afford the product (**16**) as a white solid (8 mg, 63.0%). ^1^H-NMR (300 MHz, CDCl_3_): *δ* 7.29 (5H, s, Bn), 7.24 (1H, s, H-4 of pyrazole), 5.96 (1H, s, H-5 of pyrazole), 5.28 (1H, br s, H-12), 5.12–5.02 (2H, ABq, *J* = 12.6 Hz, CH_2_ of Bn), 4.48 (1H, t, *J* = 8.1 Hz, H-3α), 4.36 (1H, dd, -N-CH_2_-CH-, *J* = 6.9, 13.5 Hz), 4.03 (1H, m, -N-CH_2_-CH-), 3.08 (1H, m, -CH_2_-CH-COOH), 2.90 (1H, d, *J* = 9.6 Hz, H-18), 2.25 (3H, s, CH_3_ of pyrazole), 1.16 (3H, d, CH_3_-CH-COOH), 1.12 (3H, s, CH_3_), 0.91 (3H, s, CH_3_), 0.89 (6H, br s, 2CH_3_), 0.82 (6H, br s, 2CH_3_), 0.60 (3H, s, CH_3_). ESI-MS: *m/z* [M + H]^+^ 697.8.

### 3.9. Synthesis of Compound ***3d***

To a stirred solution of **16** (8 mg, 0.011 mmol) in MeOH (0.5 mL) was added 10% Pd/C (1 mg) and H_2_. The mixture was stirred for 2 h at room temperature. After another portion of 10% Pd/C (4 mg) was added, the reaction was maintained for another 12 h, then filtered and the filtrate was concentrated. The residue was purified through a silica chromatography column (petroleum ether−acetone 15:1) to afford the product (**3d**) as a white solid (4 mg, 66.0%). ^1^H-NMR (300 MHz, CDCl_3_): *δ* 7.24 (1H, s, H-4 of pyrazole), 5.95 (1H, s, H-5 of pyrazole), 5.27 (1H, br s, H-12), 4.48 (1H, m, H-3α), 4.36 (1H, m, -N-CH_2_-C-), 4.04 (1H, m, -N-CH_2_-C-), 3.06 (1H, m, -CH_2_-CH-COOH), 2.82 (1H, d, *J* = 10.2 Hz, H-18), 2.25 (3H, s, CH_3_ of pyrazole), 1.16 (3H, d, CH_3_-CH-COOH), 1.13 (3H, s, CH_3_), 0.93 (6H, s, 2CH_3_), 0.90 (3H, s, CH_3_), 0.84 (3H, s, CH_3_), 0.82 (3H, s, CH_3_), 0.74 (3H, s, CH_3_). ^13^C-NMR (125 MHz, CDCl_3_): *δ* 182.9, 174.2, 148.8, 128.9, 126.8, 122.5, 104.8, 81.2, 55.3, 47.5, 46.5, 45.8, 41.6, 41.0, 39.3, 38.0, 37.0, 33.8, 33.0, 32.5, 32.4, 30.7, 28.0, 27.8, 27.7, 25.9, 23.6, 23.4, 22.9, 18.1, 17.1, 15.3, 15.1. ESI-MS: *m/z* [M − H]^−^ 605.8.

### 3.10. Assembling the Database of Carboxylic Acids

A database of all the commercial available carboxylic acids was extracted from the MDL^®^ ACD package as an .sdf file. The file was input into Accelrys Discovery Studio 2.0 (Accelrys Inc., San Diego, CA, USA) to calculate the molecular properties, such as Molecular Weight, H-bond Acceptor, H-bond Donor, etc. The output file was opened in Discovery Studio Visualizer, and the undesired molecules were filtered out manually from the file according to the criterions, which were demonstrated in the context.

### 3.11. Evaluating the Diversity of the Carboxylic Acids Database

The database file generated in the last step was input into Accelrys Discovery Studio 2.0 (Accelrys Inc., San Diego, CA, USA) to be clustered into 285 classes using a 2D fingerprint descriptor FCFP_4. One molecule was randomly selected from each cluster and put into an .sdf file to consist of a sub-library consisting of 285 members.

### 3.12. Docking

Autodock 4.2 was employed to screen a library of OA-containing structurally diverse 3-*O*-esters [29]. The crystal structure of FXR in complex with its agonist fexaramine (PDB code 1OSH) was used as the receptor. As a positive control, fexaramine was first docked against the FXR structure to validate the docking method. Docking was performed with a 60 × 60 × 60 grid covering the entire ligand binding site and 10 runs per ligand were done using the Lamarckian Genetic Algorithm (LGA) method. Other parameters were set as defaults. Top five compounds (including positive control) from the above screening were selected and further examined using Autodock Vina with default parameters [30]. To obtain more precise docking poses, a second run of Autodock was performed on these selected compounds and the parameter of LGA runs was set to 100. Docking figures were prepared using the PyMOL program (DeLano Scientific LLC: Palo Alto, CA, USA).

### 3.13. Cell Culture

293T cells were maintained in MEM media with 10% fetal bovine serum (FBS), 100 U/mL penicillin and streptomycin and 1 mM non-essential amino acid under humidified air containing 5% CO_2_ at 37 °C.

### 3.14. Transient Transfection and Luciferase Reporter Assay

293T cells were plated in 96-well plates at 2.5 × 10^4^ per well. Five ng/well pCMV-GAL4-DBD-hFXR-LBD expression vector, 25 ng/well pFRLuciferase and 5 ng/well Renilla-Luciferase reporter plasmids were transiently transfected into cells using 0.25 μL/well lipofectamine (Invitrogen, Carlsbad, CA, USA). Eighteen hours after transfection, cells were treated with CDCA at 25 μM in fresh MEM containing 0.5% charcoal-stripped FBS. Chemical derivatives dissolved in dimethyl sulfoxide (DMSO) were additionally added in concentrations indicated. Luciferase activities were measured after an additional 24 h using Dual-Luciferase Reporter Assay System (Promega, Madison, WI, USA). Relative activity was defined as pFRLuciferase activity/Renilla-Luciferase activity. The relative activity of FXR in the presence of 25 μM of CDCA was set as 100%.

## 4. Conclusions

In summary, a virtual screening strategy by combining 2D similarity search and molecular docking has been applied to the discovery of new pentacyclic triterpenoid OA-based FXR antagonists. Four OA analogs were synthesized, and two 3-*O*-ester compounds showed the improvement on FXR antagonism. These results proved the feasibility of our research strategy and the current ligand-FXR model in the further pursuit of more potent FXR interacting agents.

## Figures and Tables

**Table 1:** The predicated p*K*_i_ values and antagonistic activities of oleanolic analogs.

Prediction	1	3a	3b	3c	3d	Fexaramine
p*K*_i_ (Autodock)	8.87	8.67	9.35	8.00	9.68	10.39
IC_50_ (μM)	13.69	19.41	7.03	13.74	9.03	/

## Supplementary Materials

Supplementary materials are available online: experimental details for the synthesis of the known compounds **2a**, **2d**, **4**, **6**-**9**. Copies of ^1^H-NMR and ^13^C-NMR spectra of the novel compounds **3a**–**d**, **13**, **16**. Binding modes of OA (green) and **3a** (yellow) into FXR.
