# Supplementary material for: Discovery of Farnesoid X Receptor Antagonists Based on a Library of Oleanolic Acid 3-O-Esters through Diverse Substituent Design and Molecular Docking Methods
Source: Molecules. 2017 Apr 26;22(5):690. doi: 10.3390/molecules22050690 (PMC6154651; doi:10.3390/molecules22050690)
Supplement: Supplementary file 1 [file molecules-22-00690-s001.pdf]

Discovery of Farnesoid X Receptor Antagonists Based on a Library of 3-*O*-Esters of Oleanolic Acid through Diverse Substitution Design and Molecular Docking Methods

Shao-Rong Wang [1,2,], Tingting Xu [3,4,**] Kai Deng [1], Chi-Wai Wong [5], Jinsong Liu [4], and Wei-Shuo Fang [1,*]**

[1] State Key Laboratory of Bioactive Substances and Functions of Natural Medicines, Institute of Materia Medica, Chinese Academy of Medical Sciences & Peking Union Medical College, 2A Nanwei Road, Beijing 100050, China; wangshaorong@outlook.com

[2] Center for Drug Evaluation, China Food and Drug Administration, 1A Fuxing Road, Beijing 100038, P. R. China

[3] School of Life Sciences, University of Science and Technology of China, Hefei 230026, China; xu_tingting@gibh.ac.cn

[4] State Key Laboratory of Respiratory Disease, Guangzhou Institutes of Biomedicine and Health, Chinese Academy of Sciences, Guangzhou 510530, China; liu_jinsong@gibh.ac.cn

[5] NeuMed Pharmaceuticals Limited, Unit 509, 5/F BioTech Center I, No. 9 Science Park West Avenue, Shatin, Hong Kong; wongcw123456@yahoo.com

* Correspondence: wfang@imm.ac.cn; Tel.: +86-10-63165229

** These authors contributed equally to this paper.

Table of Contents

Experimental details for the synthesis of the known compounds 2a, 2d, 4, 6-9…………………2

Copies of ^{1}HNMR and ^{13}CNMR spectrum of novel compounds 3a-d, 13, 16……………..…..…4

Figure S1 ……………………………………………………………………………………...9

Synthesis of compound 4

To a stirred solution of **1a** (200 mg, 0.44 mmol) in a mixed solvent of dioxane (2 ml) and water (2 ml) was added K_2CO_3 (121 mg, 0.88 mmol) and tetrabutylammonium bromide (112 mg, 0.35 mmol). After benzyl chloride (100 µl, 0.88 mmol) was added dropwise, the mixture was heated to 80ºC and maintained for 3 h. The excess K_2CO_3 was filtered off. The filtrate was concentrated under reduced pressure. And the residue was purified through a silica chromatography column (petroleum ether : acetone 10 : 1) to afford the product (**4**) as white solid (198 mg, 83.5 %).

Synthesis of compound 6

To a stirred solution of **5** (106 mg, 1.0 mmol) in a mixed solvent of MeOH (0.6 ml) and water (0.3 ml) was added Na_2CO_3 (58 mg, 0.55 mmol) and hydroxylammoium chloride (70 mg, 1.0 mmol). The mixture was stirred at room temperature for 3 h. Then water (2 ml) was added, and the pH of the solution was adjusted to 2. Diethyl ether (5 ml × 3) was used to extract the product from aqueous layer. Then the organic layer was collected, dried over Na_2SO_4 and concentrated. And the residue was purified through a silica chromatography column (petroleum ether : EtOAc 15 : 1) to afford the product (**6**) as white solid (74 mg, 61.2 %). ^{1}H NMR (300 MHz, $CDCl_3$): *δ* 8.16 (1H, s, $C_6H_5N\underline{H}OH$), 7.57 (2H, m, H-2, 6 of Ph), 7.39 (3H, m, H-3, 4, 5 of Ph).

Synthesis of compound 7

To a stirred solution of **6** (20 mg, 0.17 mmol) in dry DMF (0.2 ml) was added N-chlorosuccinimide (NCS) (5 mg, 0.037 mmol). The mixture was heated to 40ºC, and two drops of 2M HCl (aq.) was added. After 10 min, the color of the solution turned to light blue. Then the reaction was moved to room temperature. After another portion of NCS (12 mg, 0.084 mmol) was added, the mixture was stirred at room temperature for 2 h. Then water (2 ml) was added, and the aqueous layer was extracted by diethyl ether (5 ml × 3). The organic layer was collected, dried over Na_2SO_4 and concentrated. And the residue was purified through a silica chromatography column (petroleum ether : EtOAc 15 : 1) to afford the product (**7**) as white solid (14.3 mg, 55.8 %). ^{1}H NMR (300 MHz, $CDCl_3$): *δ* 7.85 (2H, m, H-2, 6 of Ph), 7.44(3H, m, H-3, 4, 5 of Ph). ESI-MS: *m/z* $[M+Na]^+$ 179.0.

Synthesis of compound 8

To a stirred solution of **7** (10 mg, 0.06 mmol) in dichloromethane (DCM) (0.2 ml) was added propargyl alcohol (5 µl, 0.09 mmol) and Et_3N (13 µl, 0.09 mmol) at room temperature. Then, the mixture was heated to 50 ºC for 2 h. After the removal of the solvent, the residue was purified through a silica chromatography column (petroleum ether : EtOAc 3 : 1) to afford the product (**8**) as white solid (10 mg, 89.0 %). ^{1}H NMR (300 MHz, $CDCl_3$): *δ* 7.78 (2H, m, H-2, 6 of Ph), 7.44 (3H, m, H-3, 4, 5 of Ph), 6.56 (1H, s, H of isoxazole), 4.81 (2H, s, H of methylene). ESI-MS: *m/z* $[M+H]^+$ 176.2.

Synthesis of compound 2a

To a stirred solution of **8** (12 mg, 0.07 mmol) in acetone (0.4 ml) was added Jones' reagent (50 µl) at room temperature. Then, the mixture was stirred for 3 h. Then water (2 ml) was added, and the aqueous layer was extracted by diethyl ether (5 ml × 3). The organic layer was collected, washed with water (5 ml × 3), dried over Na_2SO_4 and concentrated. The residue was recrystallized in $CHCl_3$ to afford the product (**2a**) as white solid (12 mg, 93.0 %). ^{1}H NMR (300 MHz, $CDCl_3$): *δ* 7.97 (2H,

m, H-2, 6 of Ph), 7.61 (1H, s, H of isoxazole), 7.54 (3H, m, H-3, 4, 5 of Ph). ESI-MS: *m/z* $[M+H]^+$ 190.3.

Synthesis of compound 9

2a (13.8 mg, 0.072 mmol) was stirred with $SOCl_2$ (1 ml) at 80ºC for 6 h. Then the excess $SOCl_2$ was evaporated under reduced pressure. And the residue was used directly for reactions below without any further purification.

Synthesis of compound 2d

After the mixture of **14** (164 μl, 2 mmol), hydroquinone (5 mg, 0.05 mmol) and **15** (0.2 ml, 2.3 mmol) was stirred at 100ºC for 10 h, another portion of **15** (0.34 ml, 4 mmol) was added, and the reaction mixture was heated to 140 ºC. After another 20 h, the reaction was stopped and concentrated. The residue was recrystallized in toluene to afford product (51 mg, 81.0 % based on 129 mg recovery of substrate). ^{1}H NMR (300 MHz, $CDCl_3$): δ 7.27 (1H, d, H-4 of pyrazole, J = 1.8 Hz), 5.95 (1H, d, H-5 of pyrazole, J = 1.2 Hz), 4.32 (1H, dd, -N-C<u>H</u>$_2$-CH-, J = 6.3, 14.1 Hz), 4.10 (1H, dd, -N-C<u>H</u>$_2$-CH-, J = 5.4, 13.5 Hz), 3.04 (1H, m, -CH_2-C<u>H</u>-COOH), 2.22 (3H, s, CH_3 of pyrazole), 1.14 (3H, d, J = 7.5 Hz, C<u>H</u>$_3$-CH-COOH). ESI-MS: *m/z* $[M+H]^+$ 169.1.

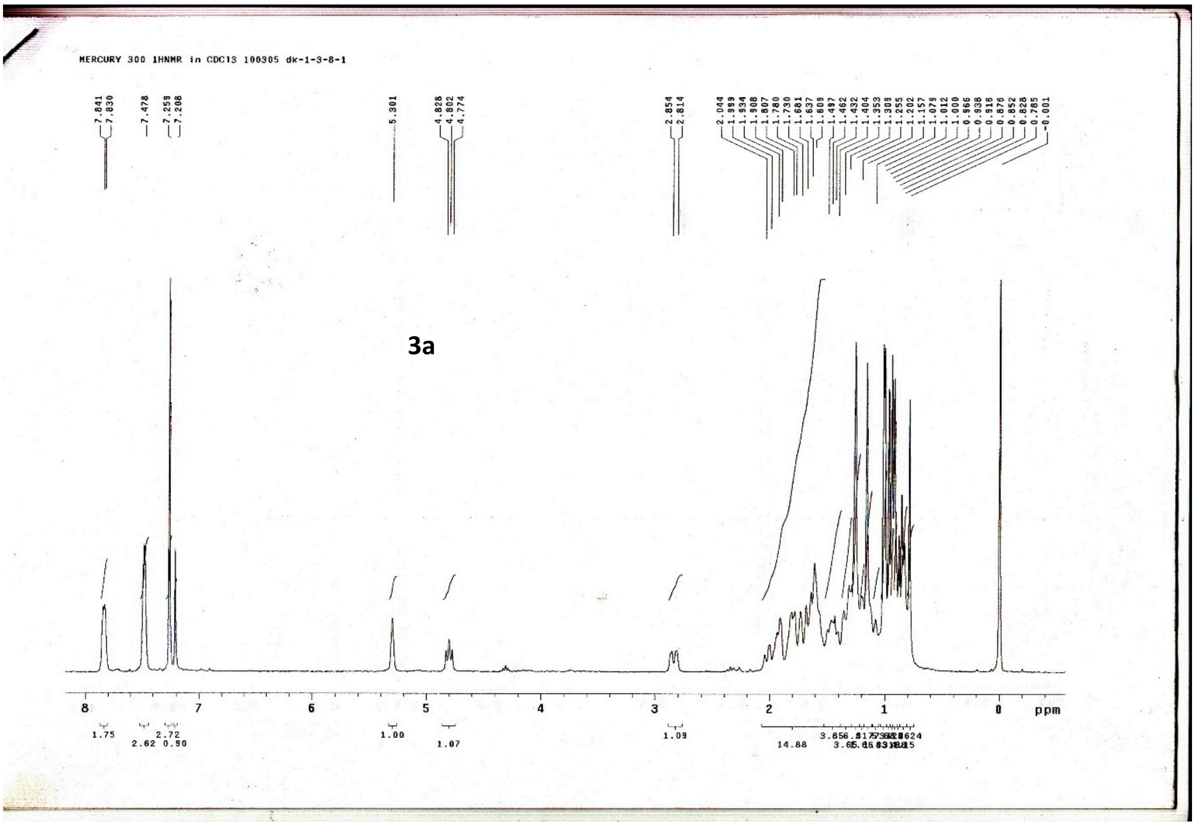
MERCURY 300 1HNMR in CDCl3 100305 dk-1-3-8-1
3a
ppm

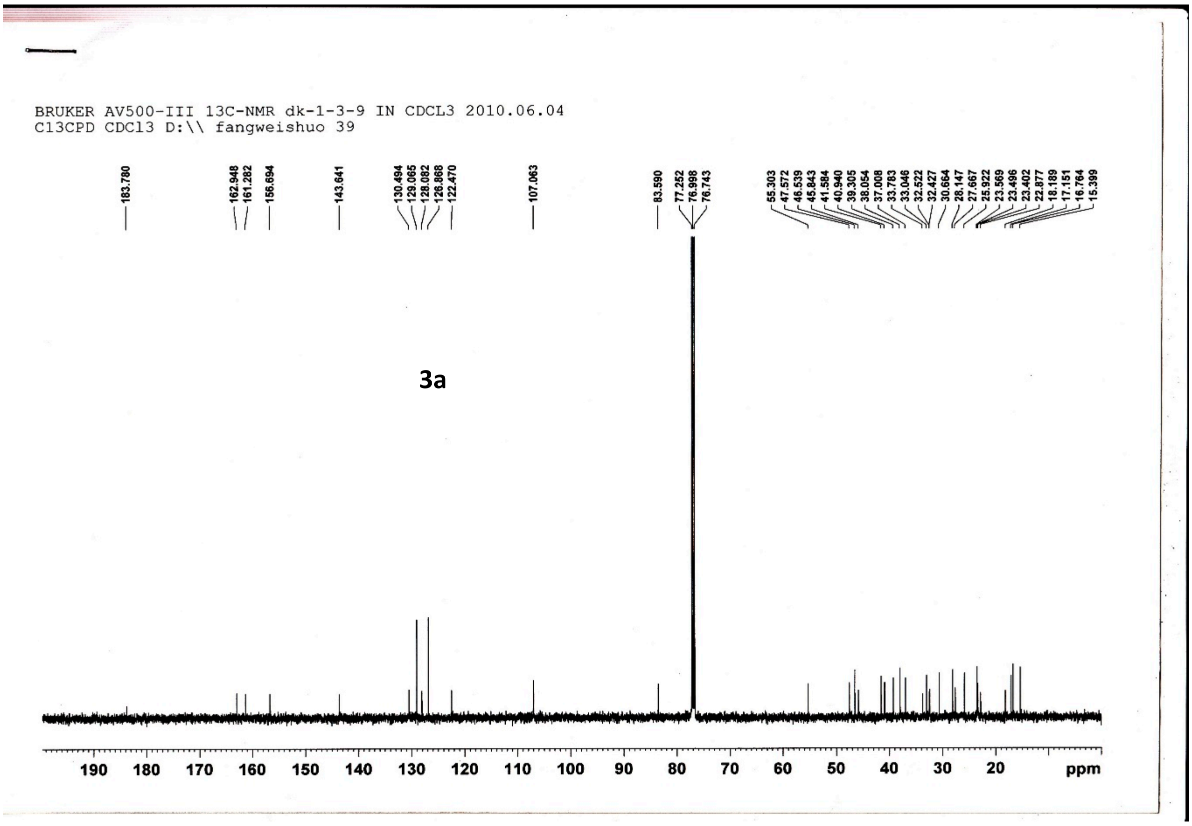
BRUKER AV500-III 13C-NMR dk-1-3-9 IN CDCL3 2010.06.04
C13CPD CDCl3 D:\\ fangweishuo 39
3a
ppm

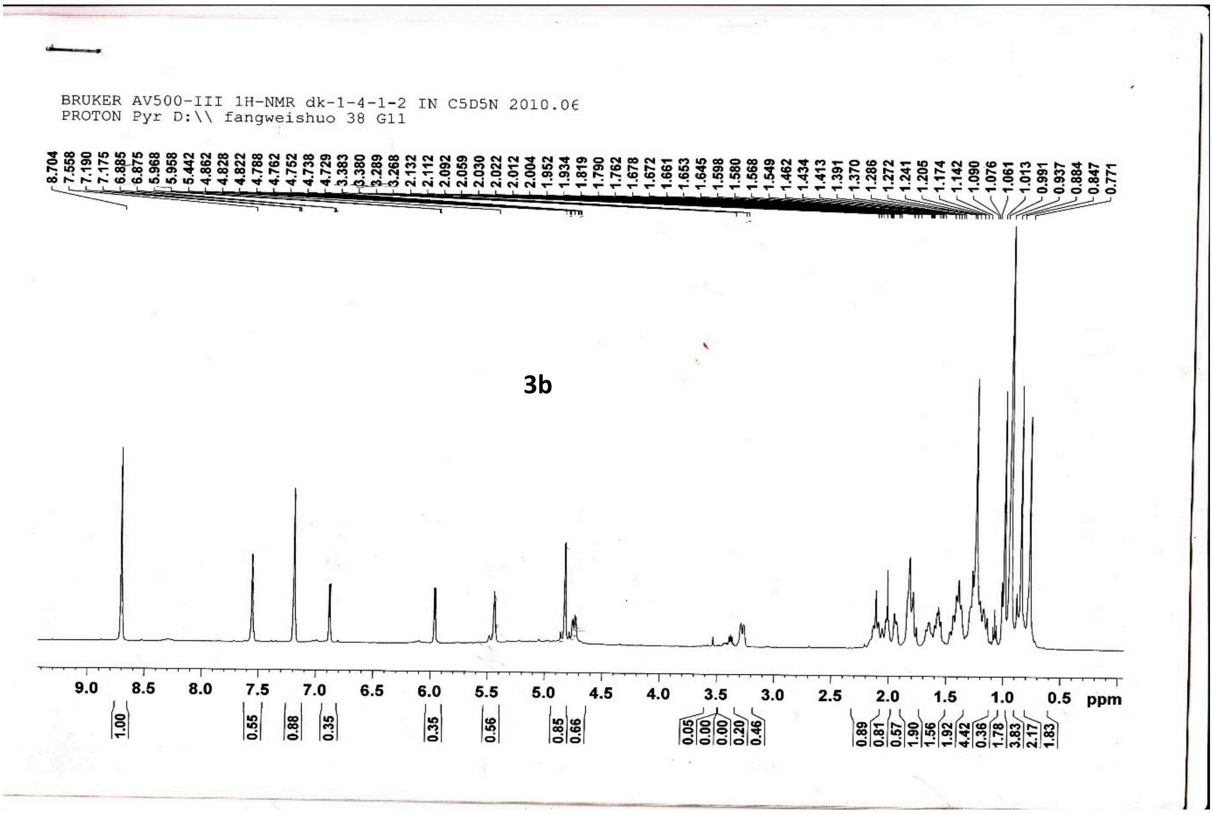
BRUKER AV500-III 1H-NMR dk-1-4-1-2 IN C5D5N 2010.06
PROTON Pyr D:\\ fangweishuo 38 G11
3b
ppm

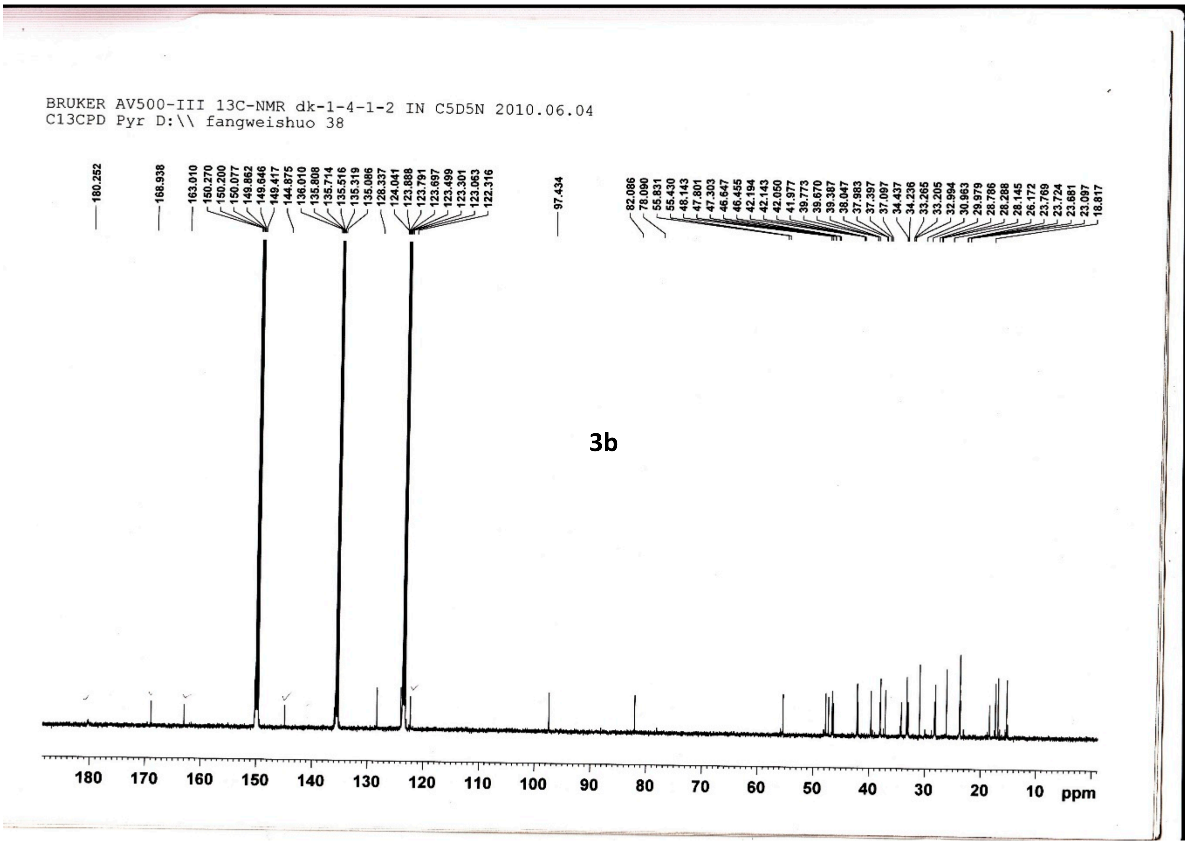
BRUKER AV500-III 13C-NMR dk-1-4-1-2 IN C5D5N 2010.06.04
C13CPD Pyr D:\\ fangweishuo 38
3b
ppm

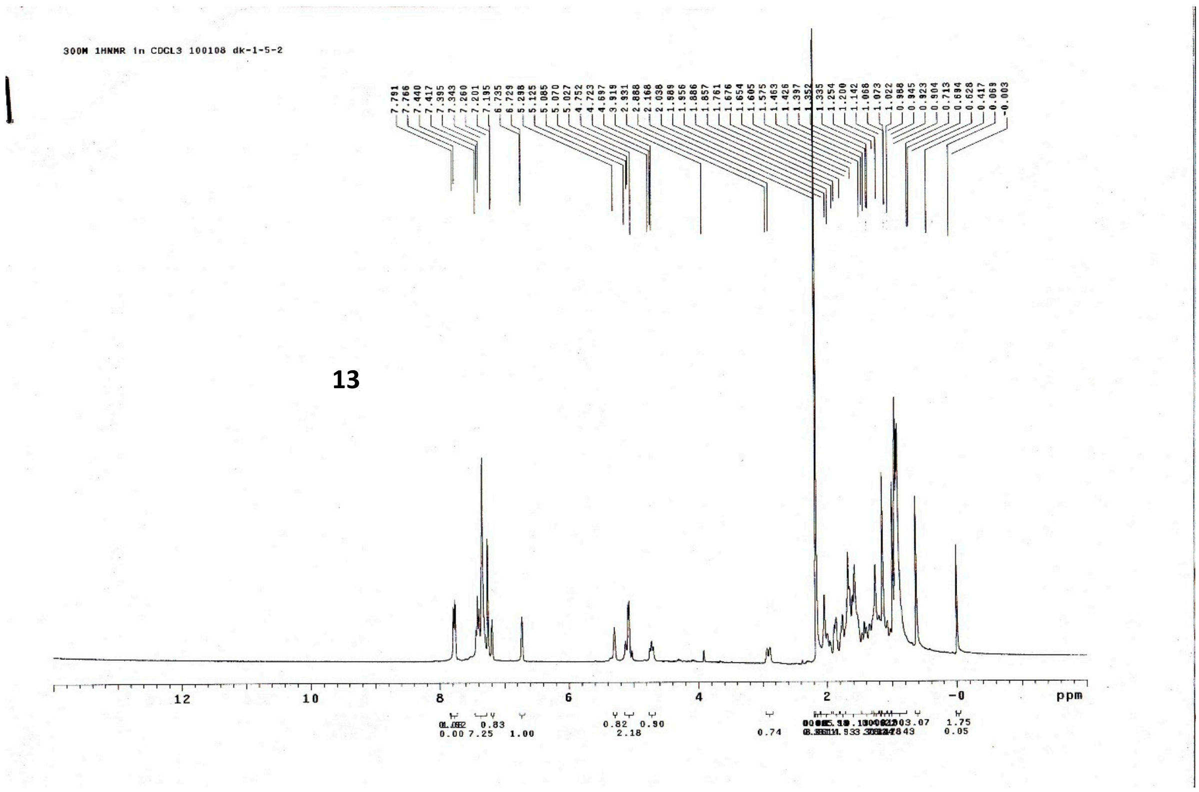
300M 1HNMR in CDCL3 100108 dk-1-5-2
ppm

13

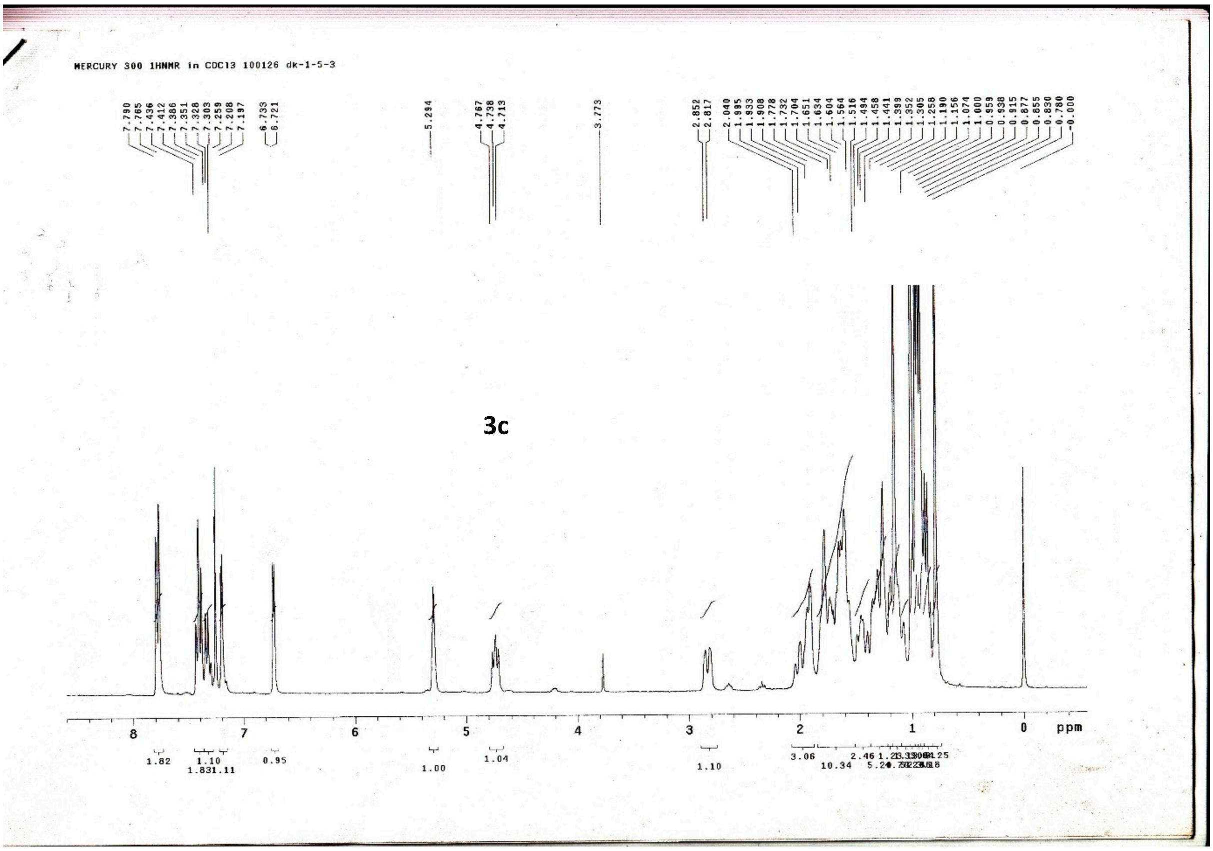
MERCURY 300 1HNMR in CDCl3 100126 dk-1-5-3
ppm

3c

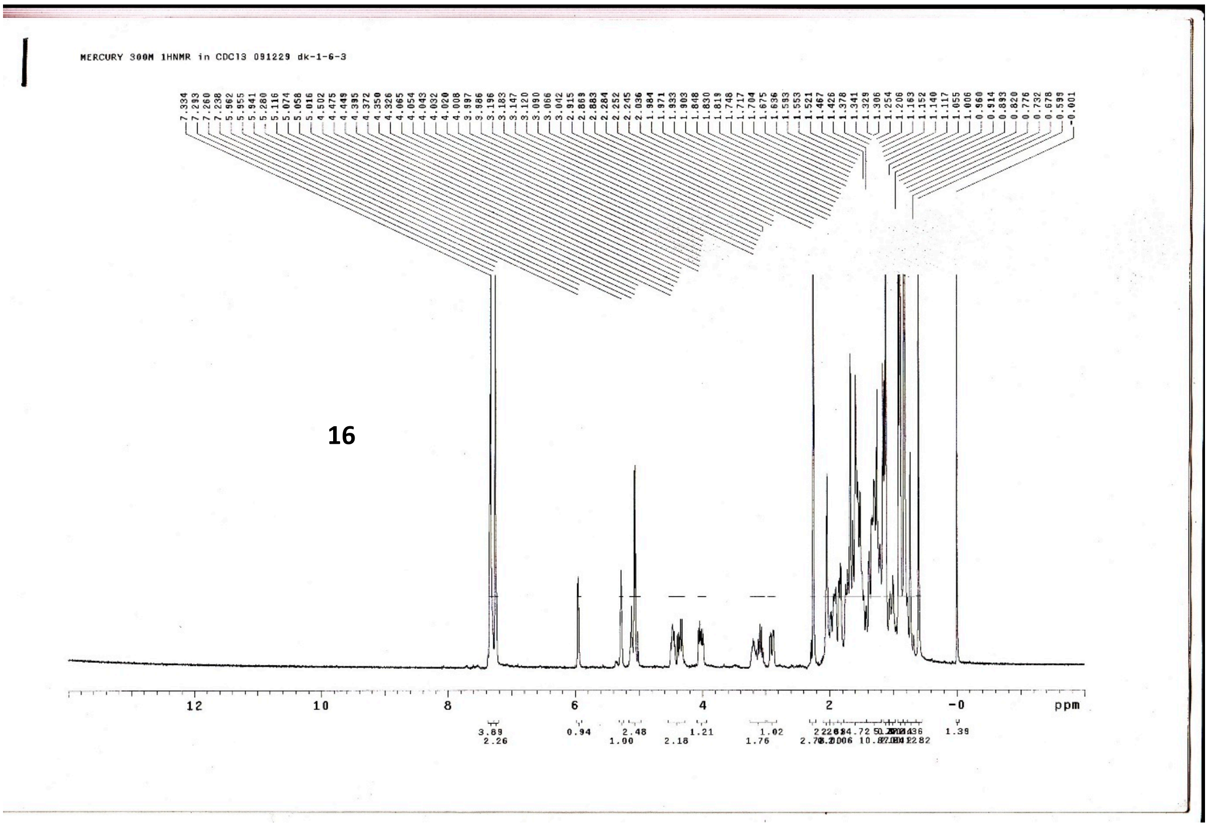
MERCURY 300M 1HNMR in CDCl3 091229 dk-1-6-3
16
ppm

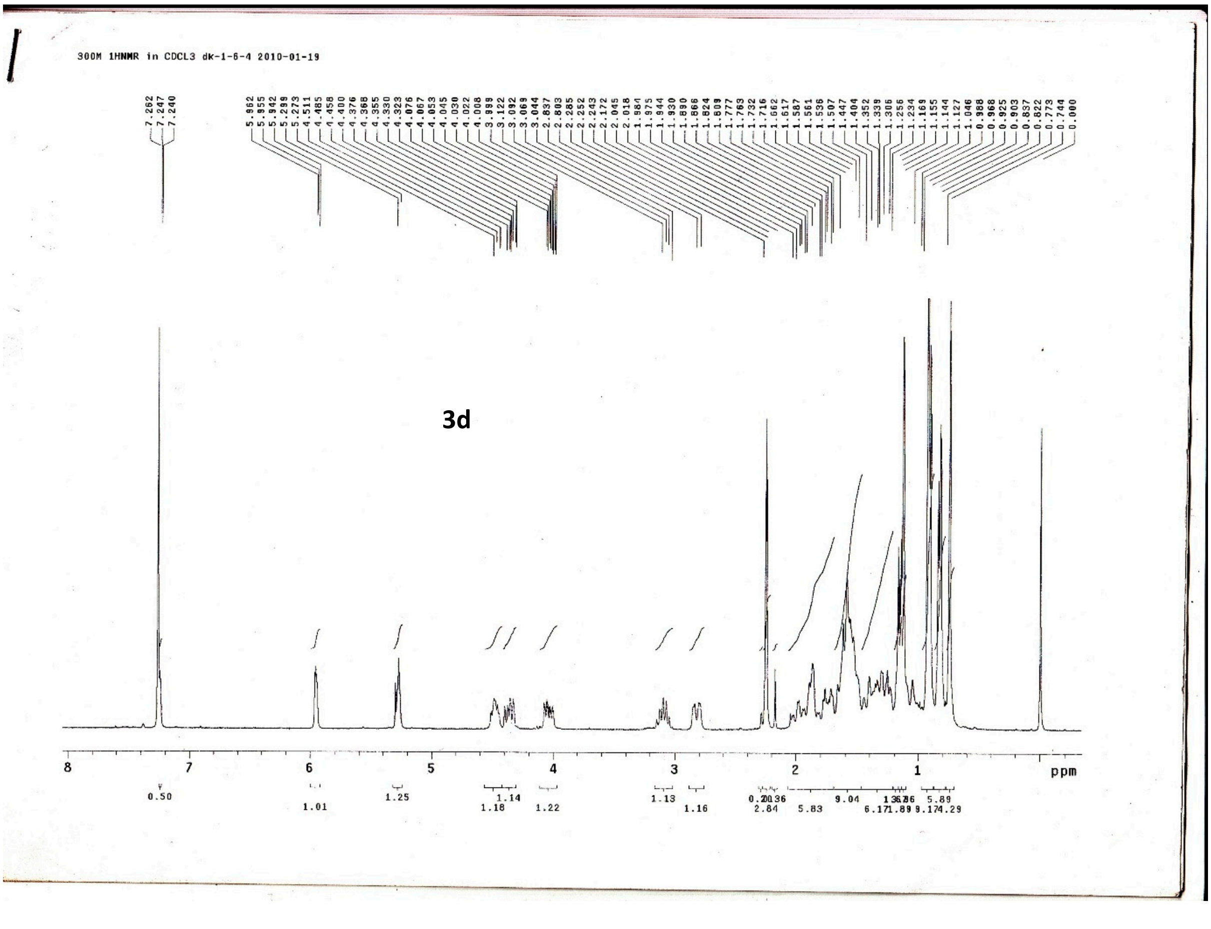
300M 1HNMR in CDCL3 dk-1-6-4 2010-01-19
3d
ppm

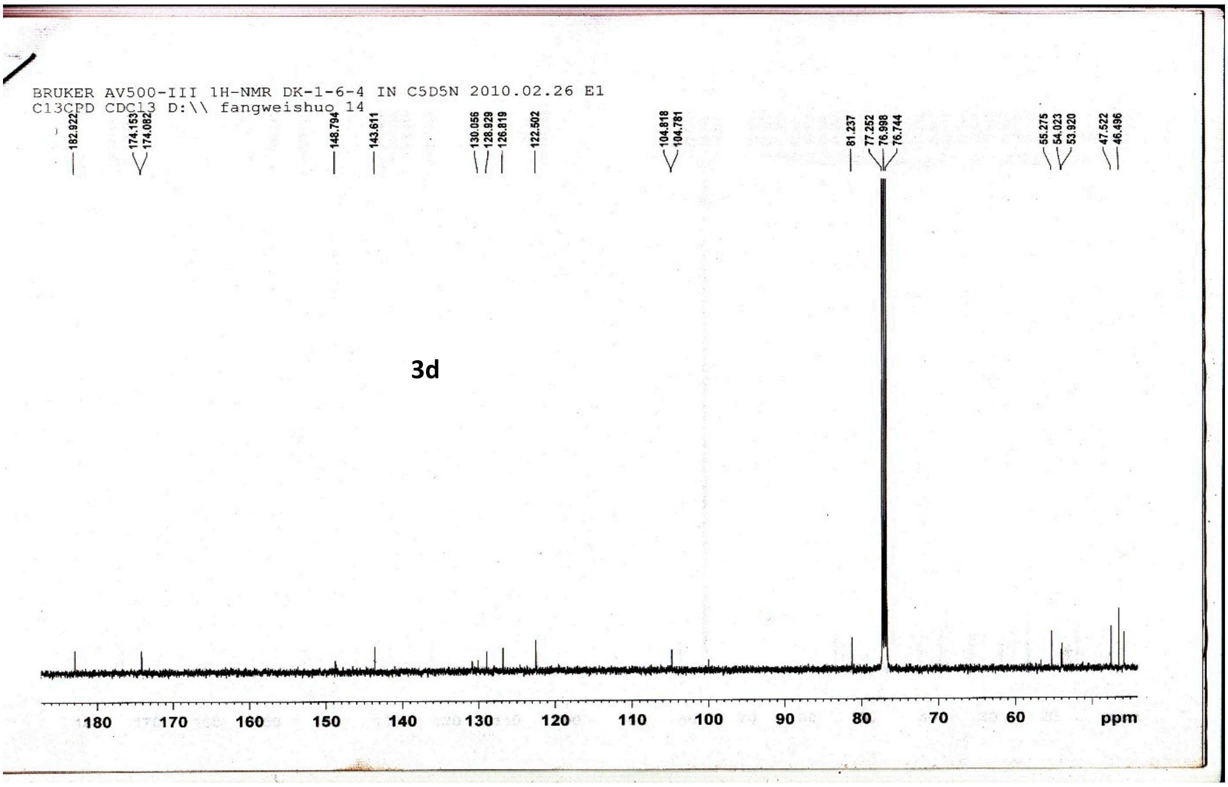
BRUKER AV500-III 1H-NMR DK-1-6-4 IN C5D5N 2010.02.26 E1
C13CPD CDCl3 D:\\ fangweishuo 14
182.922
174.153
174.082
148.794
143.611
130.056
128.929
126.819
122.502
104.818
104.781
81.237
77.252
76.998
76.744
55.275
54.023
53.920
47.522
46.496
3d
180
170
160
150
140
130
120
110
100
90
80
70
60
ppm

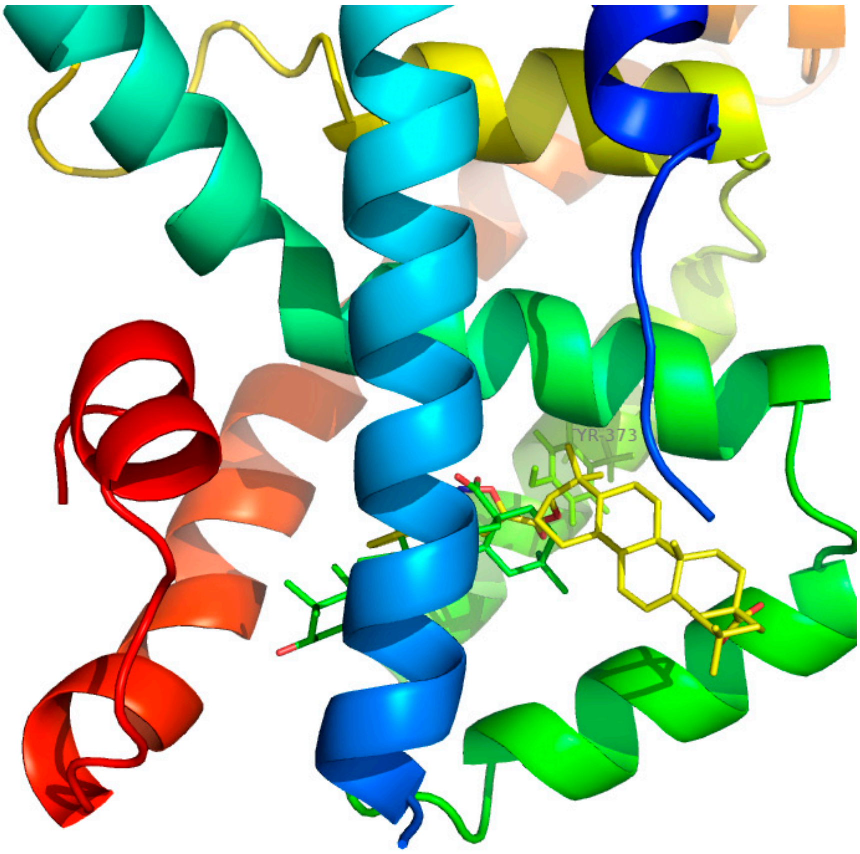

Figure S1 Binding modes of OA (green) and **3a** (yellow) into FXR
